# Tumor cell behaviour modulation by mesenchymal stromal cells

**DOI:** 10.1186/1476-4598-9-129

**Published:** 2010-05-28

**Authors:** Lucia Kucerova, Miroslava Matuskova, Kristina Hlubinova, Veronika Altanerova, Cestmir Altaner

**Affiliations:** 1 Laboratory of Molecular Oncology, Cancer Research Institute, Slovak Academy of Sciences, Bratislava, Slovakia

## Abstract

**Background:**

Human mesenchymal stromal cells (MSC) hold a promise for future cell-based therapies due to their immunomodulatory properties and/or secretory activity. Nevertheless non-neoplastic tumor compartment could also originate from MSC. We aimed to show whether multipotent MSC derived from human adipose tissue (AT-MSC) could create tumor cell-protective milieu and affect tumor cell behaviour *in vitro *and *in vivo*.

**Results:**

Here we have demonstrated tumor-promoting effect of AT-MSC on human melanoma A375 cells. AT-MSC coinjection mediated abrogation of tumor latency and supported subcutaneous xenotransplant growth from very low melanoma cell doses. Tumor incidence was also significantly increased by AT-MSC-derived soluble factors. AT-MSC supported proliferation, suppressed apoptosis and modulated melanoma cell responses to cytotoxic drugs *in vitro*. Expression and multiplex cytokine assays confirmed synergistic increase in VEGF that contributed to the AT-MSC-mediated support of A375 xenotransplant growth. Production of G-CSF and other factors implicated in formation of supportive proinflammatory tumor cell microenvironment was also confirmed. SDF-1α/CXCR4 signalling contributed to tumor-promoting effect of systemic AT-MSC administration on A375 xenotransplants. However, no support was observed for human glioblastoma cells 8MGBA co-injected along with AT-MSC that did not sustain tumor xenotransplant growth *in vivo*. Tumor-inhibiting response could be attributed to the synergistic action of multiple cytokines produced by AT-MSC on glioblastoma cells.

**Conclusions:**

Herein we provide experimental evidence for MSC-mediated protective effect on melanoma A375 cells under nutrient-limiting and hostile environmental conditions resulting from mutual crosstalk between neoplastic and non-malignant cells. This tumor-favouring effect was not observed for the glioblastoma cells 8MGBA. Collectively, our data further strengthen the need for unravelling mechanisms underlying MSC-mediated modulation of tumor behaviour for possible future MSC clinical use in the context of malignant disease.

## Background

Mesenchymal stromal cells (MSC) represent a heterogeneous population of multi-potent cells with beneficial properties for regenerative processes and/or immunomodulation [1]. Therapeutic benefit for patients suffering from a wide range of severe pathologic conditions was reported in clinical trials employing MSC and derivatives thereof [2-5]. However, MSC therapy may also bring adverse effects such as increased recurrence rate of hematologic malignancy as recently reported [6].

Increasing evidence has shown that MSC might play a role in the tumor pathogenesis and progression. Tumor behaviour is affected by non-neoplastic compartment of stroma composed from extracellular matrix, blood vessels, connective tissue, MSC, immune and inflammatory cells dynamically interlinked with tumor parenchyma [7-10]. Its growth results from the neoplastic cells' interaction with the complex stromal compartment and components thereof can be derived from progenitors residing in the bone-marrow [11,12].

Mutual cellular interactions of MSC and tumor cells were investigated in several studies to unravel the MSC effect on tumor properties. Human MSC maintained under standard culture conditions were shown to be nontumorigenic *per se*, however, several reports presented their capability to modulate tumor microenvironment thus having an impact on the tumor behaviour [13]. MSC produce cytokines with proangiogenic action, MSC can give rise to endothelial-like or pericyte-like cells contributing to tumor vasculature formation and stabilization when recruited to the site of tumor formation [12]. MSC exhibited a capability to differentiate into carcinoma-associated fibroblasts upon culture under the influence of tumor-cell produced soluble factors *in vitro *consequently leading to tumor growth support *in vivo *[14]. Unmanipulated human MSC were shown to increase the metastatic potential of breast cancer cells rather than significant tumor growth support [15]. Several other studies aimed on modelling of the interplay between tumor cells and non-tumorigenic stromal cells have shown various MSC effects on tumor cell behaviour *in vitro *and *in vivo*. MSC strongly inhibited proliferation of malignant cells of hematopoietic origin *in vitro*, nevertheless significantly increased BV173 tumor incidence *in vivo *[16]. Authors hypothesized that MSC were capable to preserve self-renewal potential of leukemic cells by mimicking cancer stem cell niche. Increased tumor incidence rather than change in tumor growth rate was reported for renal cell carcinoma, colon carcinoma and melanoma cells coinjected with MSC in syngeneic model [17]. Moreover, systemically administered MSC increased tumor incidence and allowed for the proliferation of renal carcinoma cells. Zhu *et al*. have shown similar effects of MSC-favoured tumor growth for two colon carcinoma cell lines upon coinjection with bone marrow-derived human MSC on xenogeneic model [18]. Glioma outgrowth was significantly supported by intracranial or subcutaneous tumor cell coimplantation together with human adipose tissue derived MSC [19].

On the contrary, there were several reports to show the anti-tumor effect of MSC. Khakoo *et al*. have used systemic MSC injection to inhibit the growth of Kaposi's sarcoma subcutaneous xenotransplant [20]. Prolongation of latent tumor time and tumor size decrease was shown for hepatoma cells coinjected with immortalized human fetal MSC [21]. Furthermore, MSC coimplantation with breast cancer cells resulted in inhibited tumor growth and reduced metastasis *in vivo*. [22]. Intratumoral injection of rat MSC prolonged survival in 9L glioma-bearing rats as a consequence of retarded tumor growth [23]. Several studies employing MSC as tumor-targeting delivery vehicles including our observations have reported no significant influence on tumor growth *in vivo *[24-26].

In our present study we aimed to examine the influence of human adipose tissue derived mesenchymal stromal cells (AT-MSC) on tumor development. AT-MSC could protect human melanoma cells from nutrient limitations and/or cytotoxic effects by apoptosis inhibition *in vitro*. Tumor-favouring effects on melanoma A375 xenografts were highly AT-MSC dose dependent *in vivo *and tumor incidence increase in immunocompromised host recapitulated data from syngeneic model reported previously [17]. However, AT-MSC did not increase proliferation of glioblastoma 8MGBA cells and could suppress glioblastoma 8MGBA xenograft growth *in vivo*. We hypothesized that the diversity in and responsiveness to paracrine factors produced by AT-MSC and given tumor cell lines resulted in differential tumor microenvironment composition affecting outcome of mutual tumor cell/AT-MSC interplay.

## Results

### AT-MSC support melanoma growth and increase tumor incidence *in vivo*

**Figure 1:**
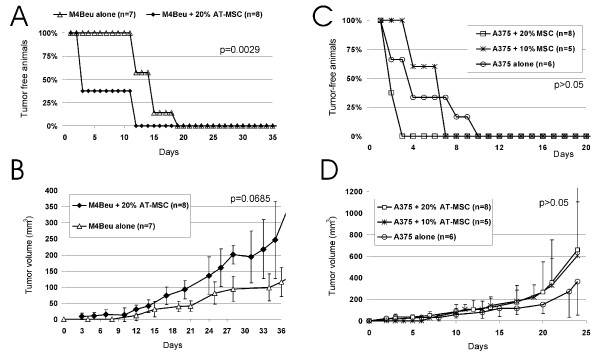
**AT-MSC coinjection with high melanoma cell dose changes the time of xenograft onset and growth**. **A.-B**. 20% AT-MSC co-administered s.c. with 1 × 10^6 ^M4Beu significantly shortened time to 100% tumor onset from day 19 (control) to day 12, but did not significantly increase tumor burden. **C.-D**. 10% or 20% AT-MSC admixed with 1.5 × 10^6 ^A375 melanoma cells also shortened time of xenograft onset and increased tumor burden in nude mice.

In order to determine, whether AT-MSC exhibit tumor supportive or inhibitory effect on melanoma cells, we first admixed AT-MSC to melanoma cell doses with 100% tumor penetrance. AT-MSC coimplanted with M4Beu melanoma cells significantly decreased time to 100%-tumor onset in comparison to M4Beu alone. Average tumor burden was higher in M4Beu/AT-MSC (ratio 5:1) group in comparison to the control group (Fig. 1A and 1B). Similarly, A375/AT-MSC (5:1 ratio) injected group of animals also exhibited shortened time to tumor onset from 10 days in A375 alone-group to 3 days concomitantly exhibiting tendency to higher average tumor volume in AT-MSC coinjected groups (Fig. 1C and 1D).

**Figure 2:**
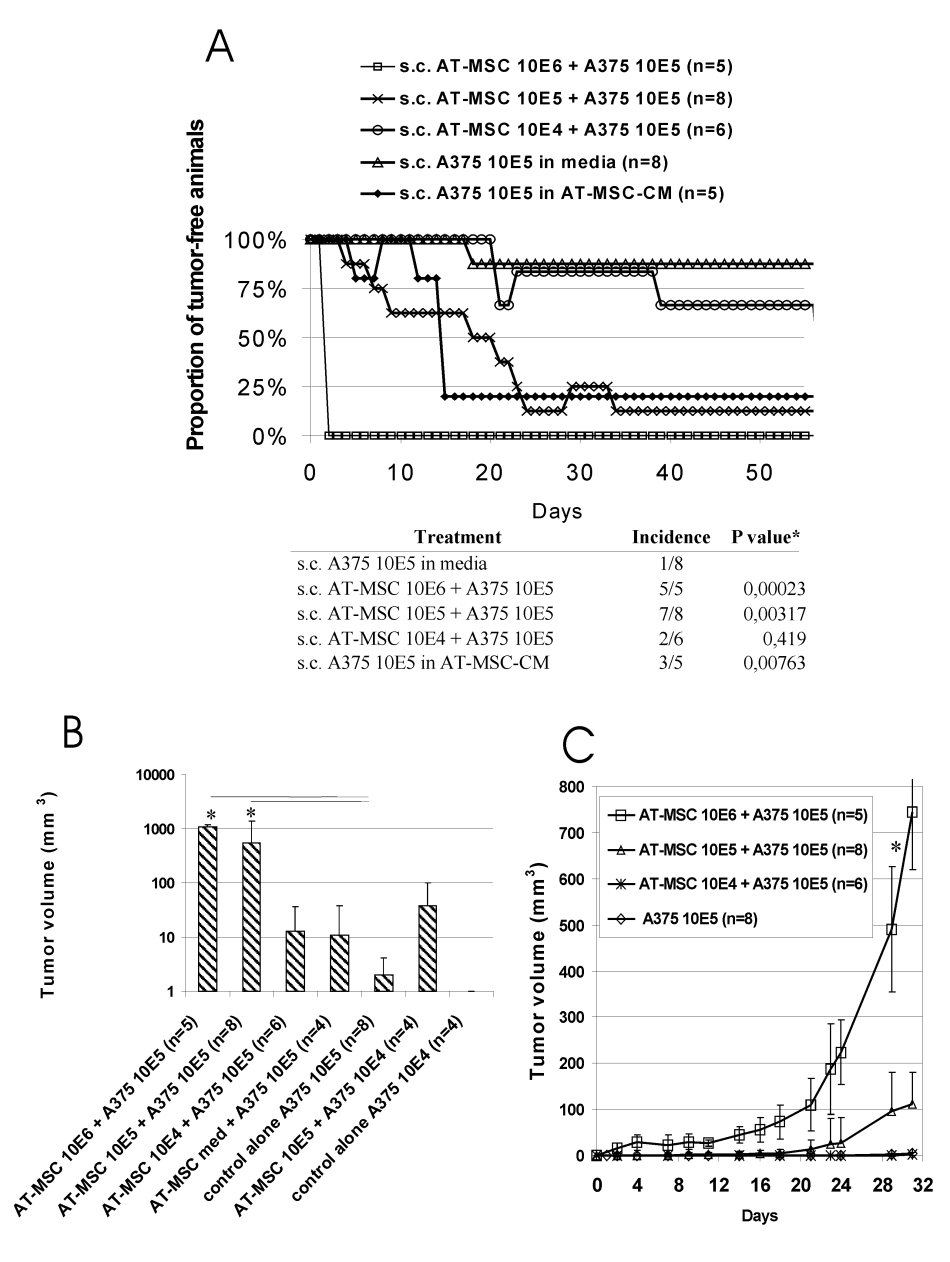
**AT-MSC abrogated tumor dormancy of low melanoma cell dose and supported tumor growth**. 1 × 10^5 ^or 1 × 10^4 ^A375 cells resuspended in serum-free culture media were injected s.c. either alone or admixed to AT-MSC in ratios 10:1, 1:1 or 1:10. Low A375 cell dose (1 × 10^5 ^s.c.) resuspended in cell-and-serum free AT-MSC-conditioned media was injected in a separate groups of animals. **A**. AT-MSC admixed to the A375 at a ratio 1:1 or 10:1 significantly increased tumor incidence in nude mice. AT-MSC conditioned media also abrogated tumor dormancy of low A375 melanoma dose. **B**. Tumor burden in 10:1 and 1:1 AT-MSC coinjected groups was significantly higher in comparison to A375 cell-induced xenografts by day 40. **C**. AT-MSC co-administration resulted in melanoma xenograft tumor growth support proportional to the AT-MSC cell dose (*P < 0.05).

Next, we aimed to determine whether AT-MSC could affect tumor incidence for limited amounts of tumor cells, which leave almost all animals long term tumor free (1 × 10^5 ^A375). AT-MSC coinjection significantly increased tumor incidence and tumor growth in groups coinjected with 10:1 or 1:1 AT-MSC to A375 cell ratio (Fig. 2A). Moreover, soluble AT-MSC produced factors were sufficient to increase tumor incidence, if AT-MSC conditioned cell-free medium was used for the A375 cell resuspension, which indicated a role of paracrine factors in tumor-promoting action. Tumor volume as a measure of tumor burden within the treatment groups was proportional to the amount of coinjected AT-MSC and significantly higher in comparison to tumor burden in control group due to earlier latency abrogation (Fig. 2B-C). Thus we conclude that AT-MSC could abrogate tumor latency for as low as 100,000 melanoma A375 cells, which would not produce tumors if injected alone in immunocompromised host.

### AT-MSC can support proliferation, protect melanoma A375 cells from nutrient deprivation or cytotoxic cellular stress *in vitro*

**Figure 3:**
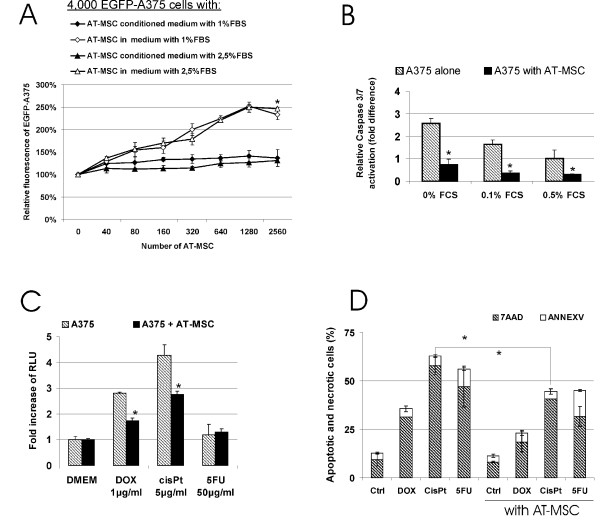
**AT-MSC increased A375 proliferation and decreased apoptosis in response to cellular stress in direct cocultures *in vitro***. **A**. Proliferation of EGFP-A375 cells when mixed with increasing numbers of AT-MSC or maintained in AT-MSC conditioned low-serum medium was evaluated by relative fluorescence after 3 days. AT-MSC significantly supported tumor cell proliferation in a dose dependent manner. This effect was significantly higher in comparison to proliferation support mediated by conditioned media from the same number of AT-MSC (*p < 0.05). **B**. A375 cells alone or mixed with 10% AT-MSC were maintained in 0%, 0.1% or 0.5% serum-containing medium for 48 hrs and relative Caspase-3/7 activation was evaluated by luminescence caspase assay. Caspase-3/7 activity of A375 cells in 0.5% serum-containing medium was set as 100%. AT-MSC significantly decreased caspase-3/7 activation in A375 melanoma cells. **C**. A375 cells alone or mixed with 10% AT-MSC were treated with doxorubicin (DOX), cisplatin (cisPt) and 5-fluorouracil (5FU) for 16 hrs under serum-deprivation conditions and Caspase 3/7 activation was evaluated by luminescence caspase assay. Results were expressed as mean increase in relative luminescence units (RLU) over background luminescence in DMEM cultured cells. AT-MSC could significantly decrease extent of caspase activation induced by doxorubicin and cisplatin in A375 cells. **D**. A375 cells alone or mixed with CFDA-SE-AT-MSC were treated with doxorubicin, cisplatin and 5-fluorouracil for 20 hrs. Proportion of apoptotic and necrotic A375 cells was determined by Annexin V and 7-AAD, respectively. AT-MSC decreased proportion of apoptotic and necrotic A375 cells thus reducing the cytotoxicity effect mediated by doxorubicin and cisplatin (*P < 0.05).

**Figure 4:**
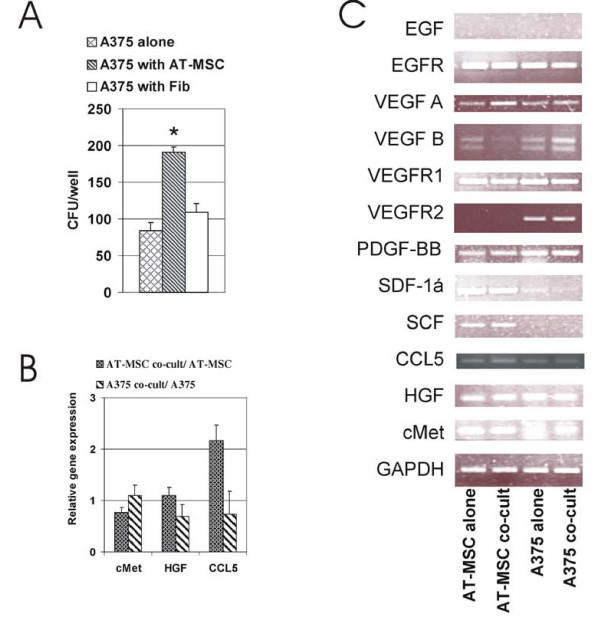
**Paracrine signalling in indirect AT-MSC/A375 co-cultures**. **A**. AT-MSC increased colony-formation by A375 cells. A375 cells were plated in the lower well part and combined with insert containing AT-MSC, fibroblast or no cells in upper part for 9 days in indirect coculture *in vitro*. Average number of A375 colonies/bottom well is shown in one representative experiment performed in triplicates (*P < 0.05). **B**. Quantitative RT-PCR was performed on templates isolated from indirectly cocultured A375 and AT-MSC cells. Gene expression level was compared to parental cells and expressed as relative gene expression. HGF and cMet expression is sustained upon coculture, CCL5 increases in AT-MSC cells cocultured with A375 cells for 3 days. **C**. Semi-quantitative expression analysis of parental A375, AT-MSC and cocultured A375 and AT-MSC cells confirms sustained expression of EGFR, VEGF A, VEGF B, VEGFR1, PDGF-bb, SDF-1α, SCF, CCL5, HGF, cMet in AT-MSC; and EGFR, VEGF-A,-B, VEGFR-1,-2 PDGF-bb, SDF-1α, CCL5, HGF, cMet in A375.

Coculture experiments *in vitro *were designed to characterize the interaction between tumor cells A375 and AT-MSC to unravel mechanism responsible for the protumorigenic effect. In order to determine the effect on tumor cell proliferation, A375 cells stably expressing EGFP (EGFP-A375) were mixed with increasing amounts of AT-MSC or conditioned medium produced from corresponding amount of AT-MSC cells. Output fluorescence was proportional to the number of EGFP-A375 cells and the amount of admixed AT-MSC cells did not interfere with output fluorescence. Soluble factors supported EGFP-A375 proliferation even in serum-limiting culture conditions although to much lesser extent in comparison to directly cocultured cells (Fig. 3A). AT-MSC also protected tumor cells from serum-deprivation induced apoptosis (A375/AT-MSC ratio 10:1, Fig. 3B). Direct cocultures of melanoma cells with AT-MSC (ratio 10:1) did not exhibit change in effector caspase activation induced by cytotoxic drugs in standard serum concentrations (not shown). However, doxorubicin and cisplatin treatment under serum deprivation conditions resulted in AT-MSC-mediated significant decrease in effector caspase-3/7 activation consequently leading to decrease in proportion of apoptotic and dead cells (Fig. 3C and 3D). Our data suggest that AT-MSC may assist tumor cells to sustain cellular stress such as nutrient deprivation and/or cytotoxicity. Indeed, in the presence of AT-MSC there was a significant increase of A375 colony-forming ability even in the absence of cell-cell contact *in vitro *(Fig. 4A). No such effect was observed when AT-MSC were added to the cultures three days later, so AT-MSC seemed to initiate colony growth at early stage. Indirect cocultures of melanoma and AT-MSC cells enabled us to analyze the expression of growth factors and receptors that was previously implicated to play a role in AT-MSC/tumor cell interactions. Quantitative analysis unravelled increased CCL5 production from AT-MSC in response to melanoma cells (Fig. 4B). Sustained expression of several potential prosurvival and proangiogenic factors and their cognate receptors in AT-MSC and A375 was demonstrated even upon 3 day coculture (Fig. 4C).

**Figure 5:**
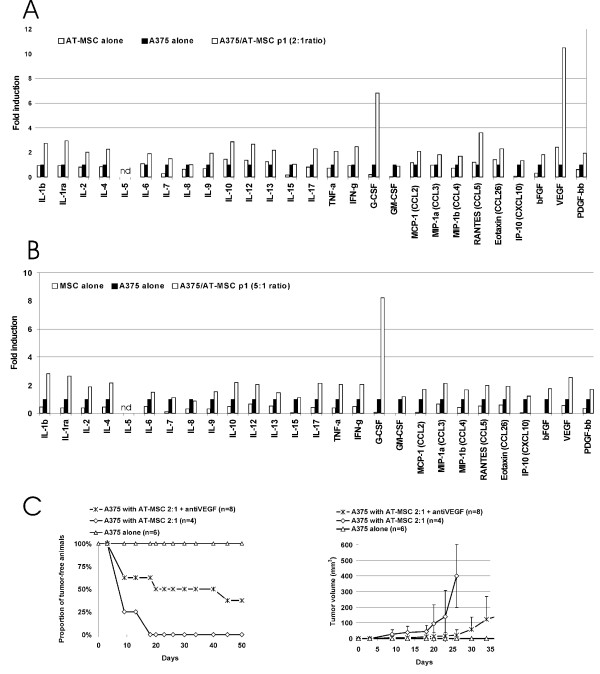
**Direct AT-MSC/A375 interactions synergistically increase VEGF production and its neutralization can partially inhibit AT-MSC mediated tumor onset and growth support**. A375, AT-MSC, or A375/AT-MSC were directly co-cultured in complete media for 3 days. A375/AT-MSC ratio was **A) **2:1 or **B) **5:1. The level of cytokines in cell-free supernatants was determined by Bio-Plex cytokine arrays and normalized to the levels observed in the media of A375 cell cultured alone. Mostly, the effects were additive or slightly synergistic. The levels of G-CSF and VEGF were significantly increased in both cases and exhibited potent synergistic effect. Data were expressed as average fold induction. ND, not detected. **C) **2 × 10^5 ^A375 cells resuspended in serum-free culture media were injected s.c. either alone or admixed to AT-MSC in ratio 2:1. One group of animals was treated with 1 mg/kg Avastin twice a week as indicated (antiVEGF group). Left panel: AT-MSC admixed to the A375 at a ratio 2:1 significantly increase tumor growth in nude mice and abrogate tumor dormancy (p = 0.0013). VEGF neutralization significantly inhibited protumorigenic AT-MSC mediated effect (p = 0.0498). Right panel: AntiVEGF treatment significantly decreased tumor burden and counteracted protumorigenic effects of AT-MSC (*p < 0.05).

Multiplex cytokine analysis was performed in order to quantitatively evaluate a paracrine signalling in tumor/AT-MSC cocultures. A wide plethora of cytokines and chemokines was detected to be secreted from both cell types. Combined coculture of these cells exhibited additive or slightly synergistic effects for most of them, nevertheless significantly increased secretion of G-CSF was detected, apparently as a response of melanoma cells, and increased VEGF production proportional to the AT-MSC number in cocultures (Fig. 5A, B). Taken together mutual crosstalk between melanoma and AT-MSC within the tumor microenvironment results in formation of proinflammatory and proangiogenic cellular milieu resulting in melanoma growth promotion *in vivo*. Next, in order to confirm relevance of the VEGF increased secretion *in vivo *we injected group of animals with mixtures of A375/AT-MSC (2:1 ratio) and treated them with neutralizing antibody against human VEGF (antiVEGF, Avastin). This treatment decreased tumor incidence in comparison to antiVEGF untreated A375/AT-MSC group to some extent and also resulted in lower average tumor burden confirming the role of VEGF in the AT-MSC mediated tumor growth support (Fig. 5C).

### Systemic administration of AT-MSC abrogates tumor dormancy and supports tumor growth via SDF-1α/CXCR4 axis

**Figure 6:**
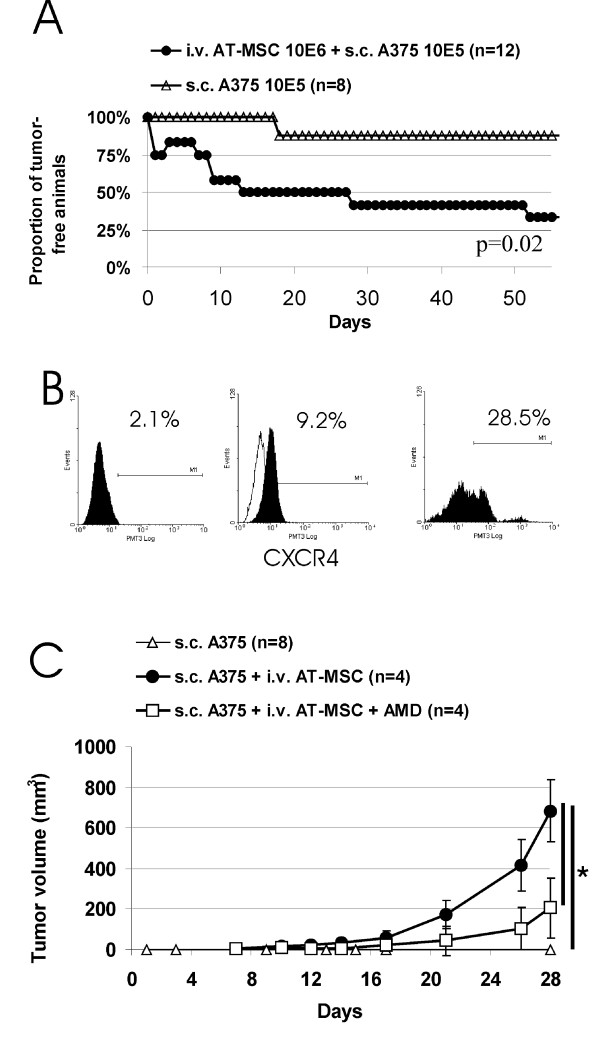
**Systemic AT-MSC administration abrogates A375 tumor dormancy and AT-MSC tumor growth support can be partially blocked by inhibiting SDF-1α/CXCR4 signalling**. **A**. Systemic administration of AT-MSC (10^6 ^i.v.) concomitant with the implantation of 1 × 10^5 ^A375 s.c. resulted in abrogation of tumor dormancy in 8 out of 12 cases in contrast to 1 out of 8 implantations of A375 s.c. alone. **B**. Cultured A375 cells or single-cell suspensions prepared by positive immunomagnetic separation of human CD44+ cells from tumor xenotransplants were stained with anti-CXCR4 antibody. Flow cytometric analysis has shown the absence of the CXCR4 marker on low density cultured A375 cells (left), CXCR4 increase upon cell confluence (middle) and high level of expression on A375 from tumor xenotransplant *in vivo *(right). CXCR4 (filled area), isotype control (open area). **C**. 2 × 10^5 ^A375 were implanted s.c either alone or coimplanted with AT-MSC (10^6 ^i.v.). Although all xenografts in AT-MSC injected group started to grow, animals treated with AMD3100 inhibitor of SDF-1α/CXCR4 (1.25 mg/kg every other day s.c.) exhibited significantly lower tumor volume in comparison to untreated group (*p < 0.05).

In order to evaluate whether AT-MSC-mediated tumor supporting effect is dependent on direct co-implantation and paracrine stimulation only, we decided to use different delivery route for AT-MSC - systemic intravenous administration frequently used in a clinical setting. AT-MSC intravenous administration concomitant with the implantation of low A375 melanoma dose s.c. (2 × 10^5^) lead to tumor growth in 7 out of 8 animals in contrast to no tumors growing without AT-MSC treatment (0/4, p = 0.00552, data not shown). Even half of the melanoma cell dose was sufficient to mediate tumor growth in 67% of AT-MSC i.v. treated animals in contrast to 12.5% A375 alone s.c. inoculations (Fig. 6A). Consistently with previously published findings [17], we were not able to detect substantial proportion of EGFP expressing AT-MSC in subcutaneous A375 xenografts post-systemic administration at experiment endpoint by flow-cytometric analysis of single-cell suspension (data not shown). This might be caused by transient and/or early AT-MSC homing at the tumor site, detection limit of the system due to the outnumbering by rapidly proliferating tumor cells and/or limited AT-MSC proliferation within the tumor xenotransplant. However, we searched for the potential key mediator(s) that could have affected early homing/incorporation of AT-MSC into xenotrasplant implantation site. Role of SDF-1α/CXCR4 axis in this process was recognized [9-11]. We have confirmed sustained SDF-1α production from AT-MSC (1,756.5 pg ± 108.2 pg per 50,000 AT-MSC). Moreover, 28.5% of A375 cells isolated from A375 xenograft expressed CXCR4 on cell surface (Fig. 6B). We hypothesized that even though the substantial amount of AT-MSC could not be detected in tumors, they actually might have homed very early into the site of tumor growth and SDF-1α/CXCR4 axis could have been responsible for the homing. SDF-1α signaling can be blocked by a CXCR4 antagonist AMD3100 - small molecule inhibitor, which enables to unravel role of this axis in the protumorigenic effects observed *in vivo*. In an attempt to abrogate tumor supportive effect of systemic AT-MSC administration on A375 xenograft, animals injected with A375s.c./AT-MSC i.v. were treated with 1.25 mg/kg AMD3100 every other day. Average tumor volume was decreased in AMD3100 treated group in comparison to control, but tumor incidences remained unaffected (Fig. 6C). These data indicated that SDF-1α/CXCR4 axis contributed to AT-MSC mediated tumor growth support; however additional mechanism(s) might be responsible for tumor dormancy abrogation upon systemic injection of AT-MSC.

### AT-MSC did not promote growth of glioblastoma cells 8MGBA *in vitro *and *in vivo*

**Figure 7:**
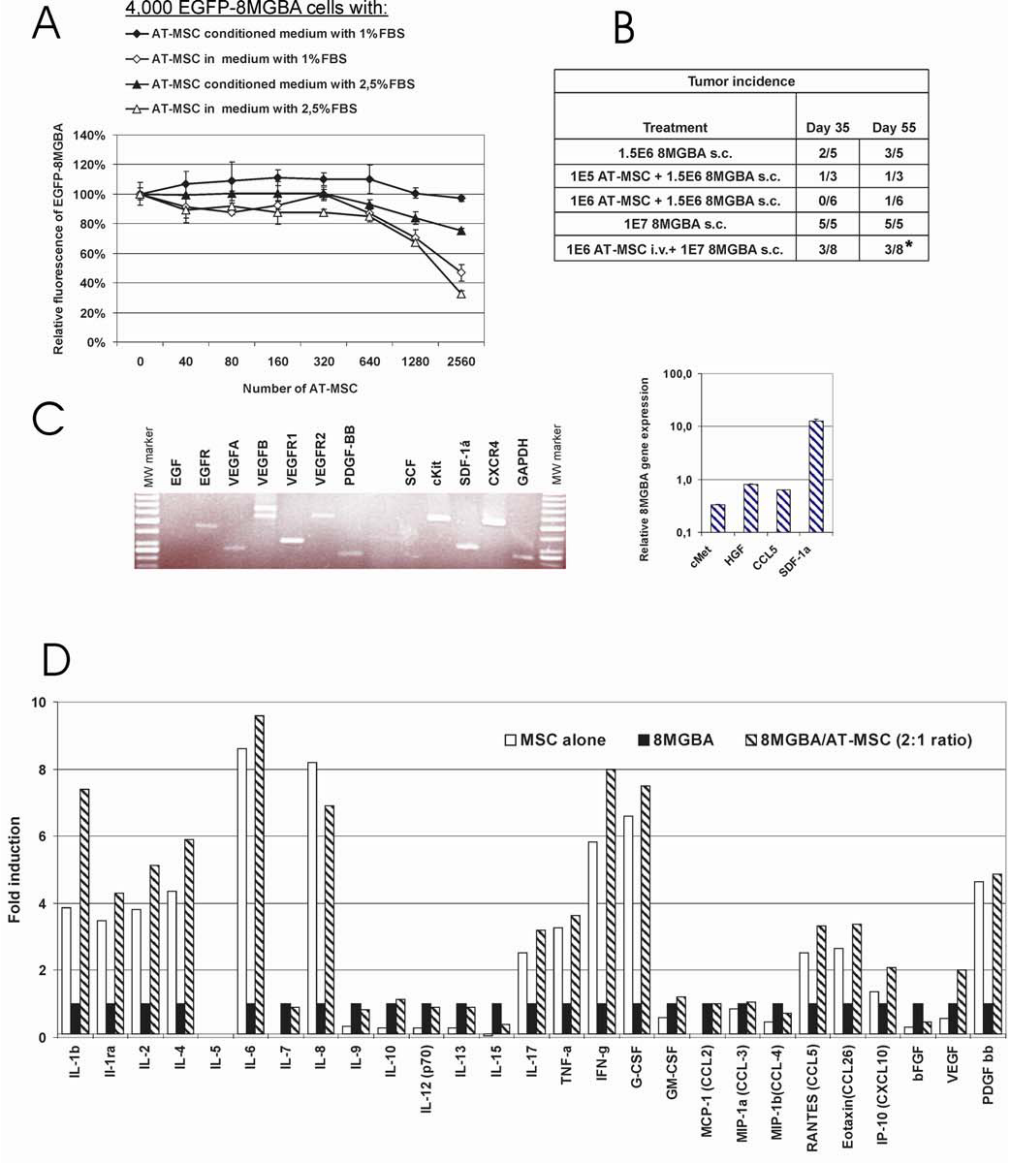
**8MGBA glioblastoma proliferation and tumor incidence was not increased by AT-MSC**. **A**. Proliferation of EGFP-8MGBA cells when admixed with increasing numbers of AT-MSC or maintained in AT-MSC conditioned low-serum medium was evaluated by relative fluorescence after 3 days. AT-MSC did not support tumor cell proliferation in comparison to control without AT-MSC. EGFP-8MGBA proliferation was significantly inhibited in co-culture containing 39% of AT-MSC (*p < 0.05). **B**. 8MGBA cells 1.5 × 10^6 ^or 1 × 10^7 ^were injected s.c. either alone, admixed to AT-MSC at a ratio 1:1 or 1:10 or 1 × 10^6 ^AT-MSC i.v. AT-MSC decreased the tumor incidence by day 55 from 60% in 8MGBA alone group to 33% in 8MGBA/AT-MSC 10:1 group and 17% in 8MGBA/AT-MSC 1:1 group. Systemic AT-MSC administration significantly decreased tumor incidence to 37.5% (*p = 0.0304). **C**. 8MGBA expression profile demonstrated expression of EGFR, VEGF-A,-B, VEGFR-1,-2 PDGF-bb, cKit, SDF-1α (high), CXCR4, CCL5, HGF, cMet (low). Quantitative differences were detected in higher level of SDF-1α expression and lower level of cMet receptor expression in comparison to A375 melanoma. **D**. 8MGBA, AT-MSC, or 8MGBA/AT-MSC (ratio 2:1) were directly co-cultured in complete media for 3 days. Level of cytokines in cell-free supernatants was determined by Bio-Plex cytokine arrays and normalized to the levels observed in the media of 8MGBA cells cultured alone. Mostly, the effects were additive, increased level of IL-1β and IFN-γ was observed in directly cocultured cells. Data were expressed as average fold induction. ND, not detected.

In order to determine whether AT-MSC have tumor supportive effect on different tumor cell type, we have performed coculture experiments *in vitro *with glioblastoma cells 8MGBA. 8MGBA cells stably expressing EGFP were mixed with increasing amounts of AT-MSC or conditioned medium produced from corresponding amount of AT-MSC cells. Output fluorescence was proportional to the number of EGFP-8MGBA cells and was not influenced by the amount of admixed AT-MSC cells. Soluble factors did not support EGFP-8MGBA proliferation and directly cocultured EGFP-8MGBA/AT-MSC cocultures exhibited proliferation inhibition at highest 8MGBA/AT-MSC proportions (Fig. 7A). Neither local coinjection nor systemic AT-MSC administration promoted glioblastoma xenograft growth *in vivo*. Implantation of high glioblastoma cell dose s.c. (10^7 ^8MGBA) concomitantly with systemic AT-MSC administration resulted in 37.5% tumor incidence by day 55 (3 out of 8), which represented significant tumor growth suppression (p = 0.0304). AT-MSC showed tendency to decrease tumor incidence upon admixing to low 8MGBA glioblastoma cells dose (Fig. 7B), even though expression analysis has shown similar expression pattern for the 8MGBA except for constitutive CXCR4 expression in comparison to melanoma cells A375 (Fig. 7C). Quantitative analysis has unravelled lower cMet receptor expression and significantly higher SDF-1α mRNA level in 8MGBA (Fig. 7C). Multiplex cytokine analysis was performed in order to quantitatively evaluate a paracrine signalling in tumor/AT-MSC cocultures. Combined coculture of 8MGBA/AT-MSC (2:1) exhibited increased secretion of IL-6, IFN-γ, G-CSF and additive effects for most of them. Overall outcome demonstrated several fold higher cytokine levels in 8MGBA/AT-MSC cocultures which might have been responsible for the observed inhibitory effect due to the synergistic action of these soluble factors (Fig. 7D).

Taken together, we have demonstrated both protumorigenic and antitumorigenic effect AT-MSC on malignant cell behaviour dependent on the mutual interplay between malignant and stromal cells to each other.

## Discussion

MSC introduction into clinical studies has brought a lot of excitement about their beneficial effect in severe pathologic situations. Anyhow, patients treated with experimental therapies have to be aware of potential unknown effects. All determinants of MSC-mediated influence on tumor behaviour have not been fully characterized so far. Discrepancies amongst several studies reflect the complexity of tumor parenchyma/non-malignant stromal cells' interplay. Adherent multipotent progenitor cells produce plethora of cytokines (Fig. 5) [27-29], that may modify tumor cell proliferation, metastasis, self-renewal, responses to cytotoxic stimuli, migration, and/or adhesion. Prosurvival action of MSC might be critical in the context of cellular stress as demonstrated in experiments performed under nutrients/cell limiting conditions *in vitro *(Fig. 3). Overall the outcome is clearly dependent on responsiveness of particular tumor cells in question and MSC-mediated changes upon interaction. Moreover, MSC physiological function implies their ability to function as niche marker cells possibly creating microregenerative niche for tumor cells that may facilitate to overcome hostile conditions *in vivo *[30,31]. Mutual tumor/MSC interplay leads to functional MSC changes even under tumor-cell produced soluble factors [14,32]. MSC do not seem to affect relative tumor growth rates *in vivo*, but substantially change tumor incidences for limiting numbers of inoculated cells [17]. Even soluble factors produced by AT-MSC were sufficient to enable the tumor (initiating) cells to overcome nutrition deprivation and engraft within the hostile microenvironment *in vivo *(Fig. 2A). Similar tumor promoting effects were reported for coinjection of fibroblasts or fibroblast conditioned medium with tumor epithelial cells supporting a role for soluble factors such as IL-6 [33-35]. These observations favour the hypothesis of MSC creating protective regenerative microenvironment by paracrine effects and/or direct interaction.

Our observations that systemic AT-MSC administration could increase melanoma xenograft incidence in immunocompromised host might be also relevant for the future clinical trials. Dormant tumors frequently present in patients do not progress into growing tumors unless angiogenic switch occurs [36]. Whether the systemic MSC administration might present such stimulus in therapeutic approaches remains to be further carefully observed. MSC incorporation into tumors can be altered by anti-inflammatory treatment thereby abrogating inhibitory effect of MSC on pancreatic tumor growth [37]. Hung *et al. *have shown MSC incorporation into established subcutaneous HT-29 xenotransplants accompanied by loss of mesenchymal concomitant with endothelial marker expression [38]. Tumor-driven differentiation of MSC into phenotype of activated fibroblasts was described as another mechanism relevant for the MSC-mediated tumor progression [14,32]. However, Djouad *et al. *could not confirm MSC incorporation into the tumor on syngeneic model and they have supported the idea of systemic immunosuppression as a mechanism facilitating increased tumor incidence in syngeneic situation *in vivo *[17].

Direct coculture of AT-MSC with glioblastoma cells has shown the absence of proliferation support *in vitro *being in line with decreased tumor incidences in 8MGBA/AT-MSC inoculations (Fig. 7). Both tumor cell types express HGF, VEGF-A and PDGF-bb - growth factors implicated in the tumor-directed MSC migration (Fig. 4C and 7C) [27,39-41]. Moreover, expression of endothelial markers such as VEGFR1 and VEGFR2 on AT-MSC suggests the AT-MSC potential to contribute to tumor vasculature and/or premetastatic niche formation [30,42]. High level of G-CSF, renowned for the promotion of survival of leukocytes and their recruitment to the tissues, was detected in tumor/AT-MSC cocultures and contributed to formation of proinflammatory microenvironment [43]. Most prominent synergistic increase of VEGF in A375/AT-MSC cocultures indicated its potential role in tumor promotion. We suggest that high local VEGF concentrations contributed to the protumorigenic action of AT-MSC, although its neutralization could not completely abrogate AT-MSC effects indicative of role of other cytokines as well (Fig. 5C). According to the previously published data, CCL5 increase could contribute to higher metastatic potential of tumor cells rather than the proliferation increase [15]. Our experimental data confirm that both local and systemic AT-MSC administration exerted protumorigenic action on A375. Intravenous AT-MSC delivery resulted in abrogation of tumor dormancy (Fig. 6A). It has been previously proposed that SDF-1α/CXCR4 signalling played role in AT-MSC homing within the sites of tumor formation and recently published data have demonstrated that inhibition of this axis by small molecule inhibitor AMD3100 could abrogate their migration towards prostate cancer cells [44]. More importantly, AMD3100 administration could abrogate glioblastoma regrowth by preventing post-irradiation recruitment of bone marrow progenitor cells [45]. Our data also demonstrate the contribution of SDF-1α/CXCR4 signalling to AT-MSC-mediated A375 tumor growth. TGFβ is another key molecule playing a role in cell recruitment and affecting expression of other chemokines thereby also modulating tumor microenvironment [46]. TGFβ signalling might be another mechanism involved in tumor dormancy abrogation as SDF-1α/CXCR4 inhibition was insufficient to counteract AT-MSC mediated effect.

It seems as though AT-MSC produced tumor-inhibitory environment for given human glioblastoma cell line 8MGBA in our study similar to reported situations of cancer inhibiting inflammatory reaction [43]. We demonstrated that these glioblastoma cells were put into relatively cytokine "rich" environment produced by AT-MSC containing IL-1β, IL-1ra, IL-2, IL-4, IL-6, IL-8, TNF-α, IFN-γ, CCL5, CCL26, CXCL10, PDGF-bb. These could act in paracrine fashion alone or in combination to suppress glioblastoma cell growth such as described for synergistic inhibitory effect of TNF-α and IP-10 (CXCL10) [47]. Indeed, Nakamura et al. reported direct antiglioma role of unmanipulated MSC resulting in prolonged animal survival upon intracranial implantation [23].

Although tumor cells were put under the influence of similar AT-MSC produced proinflammatory and proangiogenic factors, we have observed contrastingly different responses. AT-MSC presented either tumor supportive or inhibitory effects depending on the inherent tumor cell properties and response to these factors.

## Conclusions

Our study provided data to document both tumor-promoting and tumor-suppressive effects of AT-MSC on two different human tumor cell lines both *in vitro *and *in vivo*. Taken together, the complexity of tumor growth process and AT-MSC-mediated influence on tumor growth is reflected in this study. It highlights the necessity to study the tumor development in the context of tumor microenvironment to unravel determinants of tumor growth with the direct impact on therapeutic intervention [48]. Moreover, all these studies are inevitable to sufficiently describe potential MSC-attributable effects on the tumor behaviour in the light of wider MSC clinical use in both non-malignant and malignant therapeutic context.

## Methods

### Cells and Chemicals

Human melanoma cell lines A375 (ECACC No. 88113005), M4Beu, human fibroblasts (kindly provided by Dr. J. Bizik, CRI SAS, Bratislava), and glioblastoma multiforme 8MGBA (kindly provided by Dr. Perzelova, Med. School, Comenius University, Bratislava) were cultured in Dulbecco's modified Eagle medium (DMEM) supplemented with 5% fetal calf serum (FCS) and Antibiotic-Antimycotic mix (GIBCO BRL, Gaithesburg, MD). EGFP-A375 and EGFP-8MGBA cells lines stably expressing enhanced green fluorescent protein were prepared as described elsewhere [49] and cultured as above. Cells were kept in humidified atmosphere and 5% CO_2 _at 37°C. AT-MSC were derived by plastic adherence technique as previously described in [24]. Briefly, AT-MSC cells were expanded from adherent cells obtained from stromal-vesicular fraction upon collagenase type VIII digestion of lipoaspirate obtained from healthy persons, who provided an informed consent. Cells were expanded in low glucose (1,000 mg/ml) DMEM supplemented with 10% Mesenchymal stem cell stimulatory supplement (human, MSCSS) (StemCell Technologies, Grenoble, France) and Antibiotic-antimycotic (GIBCO BRL, Gaithesburg, MD). AT-MSC were characterized by surface marker expression as CD44+, CD73+, CD90+, CD105+, CD14-, CD34-, CD45- population and AT-MSC were capable of differentiation into adipocytes and osteoblasts. Conclusions were drawn from similar results of experiments performed with two different isolates if not specified otherwise.

Cell-free AT-MSC conditioned medium was collected from 80% confluent AT-MSC cultures maintained in serum-free DMEM for 24 hrs and used for inoculations *in vivo*.

All chemicals were purchased from Sigma (St. Louis, MO) if not stated otherwise.

### Tumor cell and AT-MSC cocultures

For proliferation evaluation, triplicates of 4,000 EGFP-A375 or EGFP-8MGBA tumor cells/well were seeded in black-walled 96-well plates (Greiner Bio-One Intl. AG) with or without increasing numbers of AT-MSC for overnight. Same amounts of AT-MSC in triplicates were seeded in parallel 96-well plates in 1% and 2.5% FBS containing DMEM for preparation of AT-MSC conditioned media. This media after overnight incubation was transferred into corresponding wells to evaluate tumor cell proliferation in AT-MSC conditioned medium. Medium was replaced every day and relative proliferation was evaluated by PolarStar OPTIMA reader (BMG Labtechnologies, Offenburg, Germany) on day 3. Values were expressed as means of relative fluorescence ± SD, where EGFP tumor cell fluorescence in appropriate medium DMEM without AT-MSC was set to 100% by default. It was previously determined, that there was linear correlation between fluorescence intensity and number of EGFP expressing cells under these experimental culture conditions.

For apoptosis evaluation, quadruplicates of 15,000 tumor cells/well were seeded either alone or into wells containing 1,500 AT-MSC/well in 96-well plates for overnight. Cells were washed and treated with 0%, 0.1% and 0.5% serum containing media for 3 days to determine the extent of serum-deprivation induced apoptosis. Cells were treated with 1 μg/ml doxorubicin, 5 μg/ml cisplatin or 50 μg/ml 5-fluorouracil in serum-free or 5% serum containing media for 16 hrs to evaluate extent of cytotoxicity. Caspase-3/7 activation was determined by Caspase-Glo^® ^3/7 Assay (Promega, Madison, WI) on LUMIstar GALAXY reader (BMG Labtechnologies, Offenburg, Germany). Values were expressed as fold increase in relative luminescence units (RLU) in comparison to tumor cells maintained in media alone.

For flow cytometric determination of apoptotic and necrotic cell proportions in direct cocultures, adherent AT-MSC were labelled with 5 μM carboxy-fluorescein diacetate, succinimidyl ester (CFDA-SE, Molecular Probes, Eugene, OR) in a serum-free DMEM for 15 min at 37°C. Medium was replaced for standard culture medium for overnight incubation. 100,000 tumor cells/well were seeded with or without 10,000 CFDA-SE-AT-MSC/well in duplicates in 24-well plates and serum-deprived for overnight. Cells were treated with 200 ng/ml doxorubicin, 5 μg/ml cisplatin or 50 μg/ml 5-fluorouracil in 5% serum containing media for 20 hrs. Apoptotic cells were stained with Phycoerythrin-labelled Annexin V (Invitrogen, Carlsbad, CA); dead cells were detected with 7-AAD viability dye. Stained cells were analyzed using an EPICS ALTRA flow cytometer (Beckman Coulter, Fullerton, CA) equipped with Expo 32 program.

Colony-formation ability was evaluated in indirect tumor cell coculture with AT-MSC or fibroblasts. Tumor cells (280/cm^2 ^in 6-well plates) were plated into wells and combined with AT-MSC or fibroblasts physically separated in upper compartment and seeded on 0.4 μm cell culture inserts (5 × 10^4 ^cell/insert) (Nalge Nunc International, Rochester, NY). Cells were maintained for 9 days in standard culture media. Average total number of A375 colonies per well was counted after Giemsa-Romanowski staining. Conclusions were drawn from three independent experiments.

### Analysis of gene expression

Tumor cells were cultured with or without AT-MSC seeded on 0.4 μm inserts (10^5 ^cells/insert) (Nalge Nunc International, Rochester, NY) for 3 days. Total RNA was isolated from 0.5 × 10^6 ^8MGBA, A375, AT-MSC, A375 co-cultured cells and AT-MSC co-cultured cells collected from inserts by RNeasy mini kit (Qiagen, Hilden, Germany) and treated with RNase-free DNase (Qiagen, Hilden, Germany). RNA was reverse transcribed with RevertAid™ H minus First Strand cDNA Synthesis Kit (Fermentas, Hanover, MD). 200 ng of cDNA was subject to standard PCR performed in 1× PCR Master Mix (Fermentas, Hanover, MD) with 35 cycles and gel resolved on 2% agarose or 4% MetaPhor^® ^Agarose (Lonza, Rockland, ME, USA) for qualitative analysis.

Quantitative PCR was performed in 1× ABsolute™ QPCR SYBR^® ^Green Mix (ABgene, Surrey, UK), 0.16 μM primers and 500 ng of template cDNA on RotorGene 2000 (Corbett Research, Sydney, Australia) and analyzed by RotorGene Software version 4.6. Primer sequences were used as previously published [15,27]. Relative gene expression change was calculated according to the formula Fold increase = (reaction efficiency*2)^ΔΔCt^, where ΔΔCt = [{Ct_GOI(control cells)_-Ct_GAPDH(control cells)_}-{Ct_GOI(treated cells)_-Ct_GAPDH(treated cells)_}]. GAPDH expression was taken as endogenous reference gene (GOI = gene of interest). Analysis was performed twice in triplicates and data expressed as means ± SE.

### CXCR4 expression

CXCR4 surface expression was analyzed in A375 cultures, or A375 cells isolated from tumor xenografts. Tumors were treated with 0.15% collagenase VIII, and 0.05 mg/ml DNAse I for 45 min at 37°C. Cells were sieved through 30 μm pre-separation filters (Miltenyi Biotec, Bergisch Gladbach, Germany) to obtain single-cell suspension and immunomagnetically separated by EasySep human FITC selection kit (StemCell Technologies, Vancouver, BC, Canada) by positive selection with FITC conjugated human specific anti-CD44 antibody (Millipore, Billerica, USA). CD44 positive cells were stained with PE-conjugated anti CXCR4 antibody (Invitrogen, Carlsbad, CA), anti-IgG2a isotype control (Invitrogen, Carlsbad, CA) and by flow cytometer. Representative result out of three independent experiments is shown.

### Cytokine secretion analysis

50,000 A375 or 8MGBA, 25,000 AT-MSC cells alone plated in wells, 10,000 AT-MSC cells alone, 50,000 A375 (or 8MGBA) cells mixed with AT-MSC (ratio 5:1 or 2:1) were cultured in complete culture medium for three days. Cell-free supernatants were collected and subjected to human Bio-Plex™ 27-plex Cytokine Assay (Bio-Rad Laboratories Inc, Hercules, CA). Measurements were performed on Luminex 100 System (Luminex Corporation, Austin, TX) in duplicates with two different AT-MSC isolates. Results were expressed as means and relative cytokine expression was calculated by comparison to cytokine production from A375 (8MGBA) cells respectively.

SDF-1α level was determined in cell free supernatants prepared as above by human SDF1-α Quantikine Immunoassay (R&D Systems Inc.) on PolarStar OPTIMA reader (BMG Labtechnologies, Offenburg, Germany) as recommended by manufacturer.

### Experiments in vivo

Six weeks old athymic nude mice (Balb/c-nu/nu) were used in accordance with institutional guidelines under the approved protocols. It was determined in preliminary studies that 10^6 ^M4Beu cells, 1.5 × 10^6 ^A375 cells or 10^7 ^8MGBA cells injected s.c. exhibit 100% tumor incidence. Following cell suspensions were injected in high dose coinjection studies: 1.5 × 10^6 ^A375 cells, 1.5 × 10^6 ^A375 + 1.5 × 10^5 ^AT-MSC (10%AT-MSC), 1.5 × 10^6 ^A375 + 3 × 10^5 ^AT-MSC (20% AT-MSC), 1 × 10^6 ^M4Beu cells, 1 × 10^6 ^M4Beu + 1 × 10^5 ^AT-MSC (10% AT-MSC) (in 100 μl of PBS s.c. into the flank). In an independent study animals received low tumor cell dose (tumor incidence 1/10) of 1 × 10^4^, 1 × 10^5 ^or 2 × 10^5 ^A375 cells s.c as indicated. Groups of animals were directly coinjected with 1 × 10^6^, 1 × 10^5^, 1 × 10^4 ^AT-MSC in admixture s.c., or s.c administered 1 × 10^5 ^tumor cells were resuspended in AT-MSC conditioned media prior to injection. Independent group of animals was systemically administered with 1 × 10^6 ^AT-MSC i.v. into the lateral tail vein concomitantly with s.c. administration of A375 cells alone. Animals were subsequently treated with 1.25 mg/kg AMD3100 every other day s.c. or 1 mg/kg Avastin (Bevacizumab, Roche, kindly provided by National Cancer Institute, Bratislava) i.p. twice a week where indicated.

For glioblastoma xenograft study animals received low (1.5 × 10^6^) 8MGBA cell dose cells s.c. Experimental groups of animals were directly coinjected with 1 × 10^6 ^or 1 × 10^5 ^AT-MSC in admixture s.c. Independent group of animals was systemically administered with 1 × 10^6 ^AT-MSC i.v. into the lateral tail vein concomitantly with s.c. administration of 8MGBA high cell dose (10^7^).

Animals were regularly inspected for tumor incidence and designated tumor-free when no palpable rigid structures exceeding 1 mm in diameter could have been detected. Growing tumors were measured by calliper and volume was calculated according to formula volume = length × width^2^/2. Animals were sacrificed at the point, when the tumors exceeded 1 cm in diameter. Results were evaluated as mean volume ± SE.

### Statistical analysis

Student's t test was used for comparison between the groups, differences in tumor incidences were evaluated by log-rank test, P value < 0.05 was considered significant.

## List of abbreviations

bFGF: basic fibroblast growth factor; cMet: hepatocyte growth factor receptor; CXCR4: SDF-1α (CXCL12) receptor; EGF: epidermal growth factor; EGFR: epidermal growth factor receptor; FBS: foetal bovine serum; GAPDH: glyceraldehyde 3-phosphate dehydrogenase; G-CSF: granulocyte-colony stimulating factor; GM-CSF: granulocyte monocyte-colony stimulating factor; HGF: hepatocyte growth factor; IFN-g: interferon γ; IL: interleukin; MCP-1 (CCL2): monocyte chemoattractant protein-1, chemokine CCL2; MIP-1a (CCL3): macrophage inflammatory protein-1alpha; MIP-1b (CCL4): macrophage inflammatory protein-1beta; PDGF-bb: platelet-derived growth factor; RANTES (CCL5): Regulated on Activation, Normal T-cell Expressed and Secreted, chemokine CCL5; SCF: stem cell factor; SDF-1α: stroma-derived factor 1α, chemokine CXCL12; TNF-a: tumor necrosis factor α; VEGF: vascular endothelial growth factor; VEGFR: vascular endothelial growth factor receptor.

## Competing interests

The authors declare that they have no competing interests.

## Authors' contributions

LK designed study, performed experiments, wrote and drafted manuscript, MM performed flow cytometry analysis and EGFP cells, KH participated on in vivo experiments, VA isolated and established AT-MSC cultures, CA provided grant support, coordination and mentorship. All authors have read and approved the final manuscript.
